# Supplementary material for: Relative genomic impacts of translocation history, hatchery practices, and farm selection in Pacific oyster Crassostrea gigas throughout the Northern Hemisphere
Source: Evol Appl. 2020 Apr 17;13(6):1380–99. doi: 10.1111/eva.12965 (PMC7359842; doi:10.1111/eva.12965)
Supplement: Supplementary file 2 — Appendix S1 [file EVA-13-1380-s002.pdf]

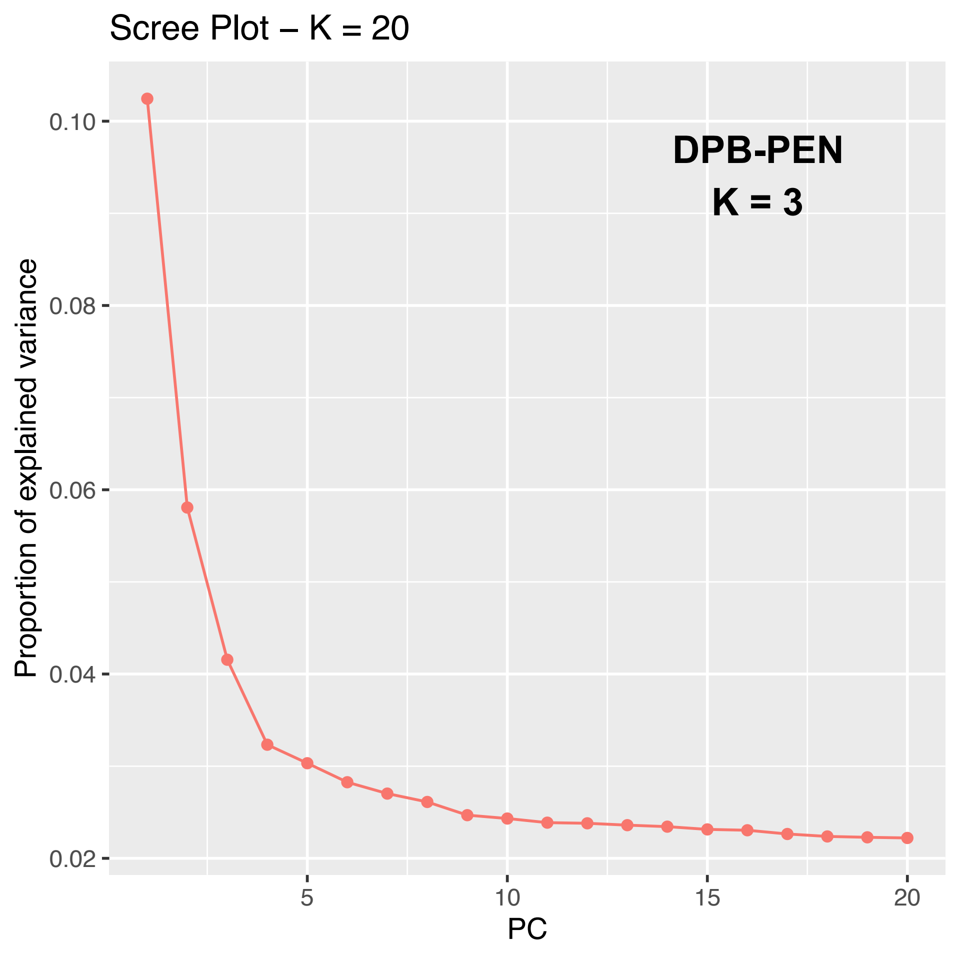
Scree Plot – K = 20
DPB-PEN
K = 3
Proportion of explained variance
0.10
0.08
0.06
0.04
0.02
PC
5
10
15
20

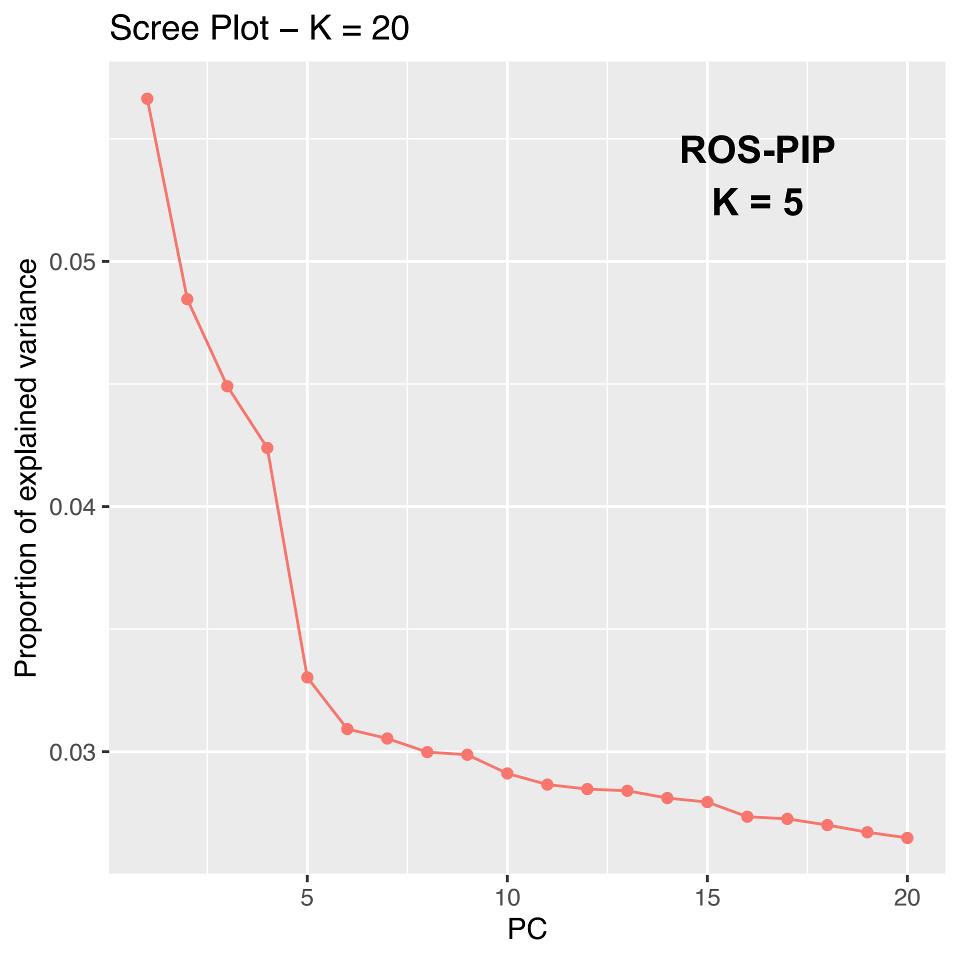

Scree Plot – K = 20
ROS-PIP
K = 5
Proportion of explained variance
0.05
0.04
0.03
5
10
15
20
PC

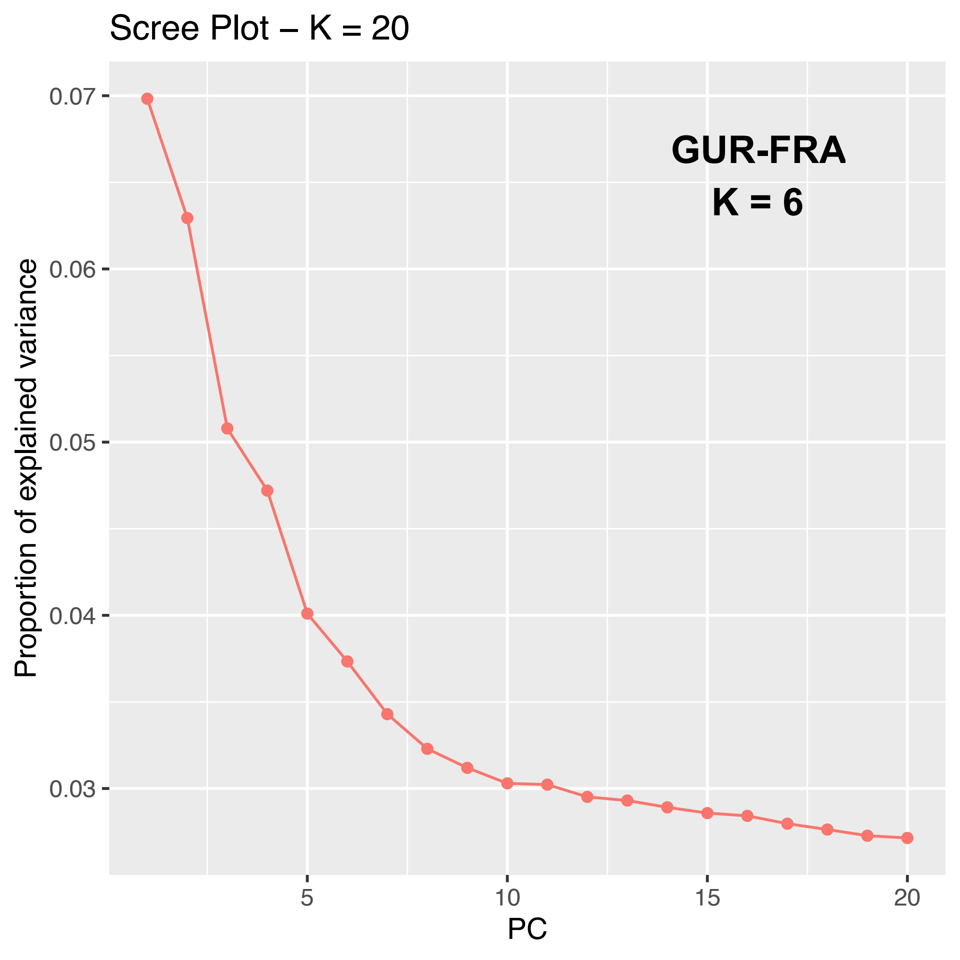
Scree Plot – K = 20
GUR-FRA
K = 6
Proportion of explained variance
0.07
0.06
0.05
0.04
0.03
PC
5
10
15
20

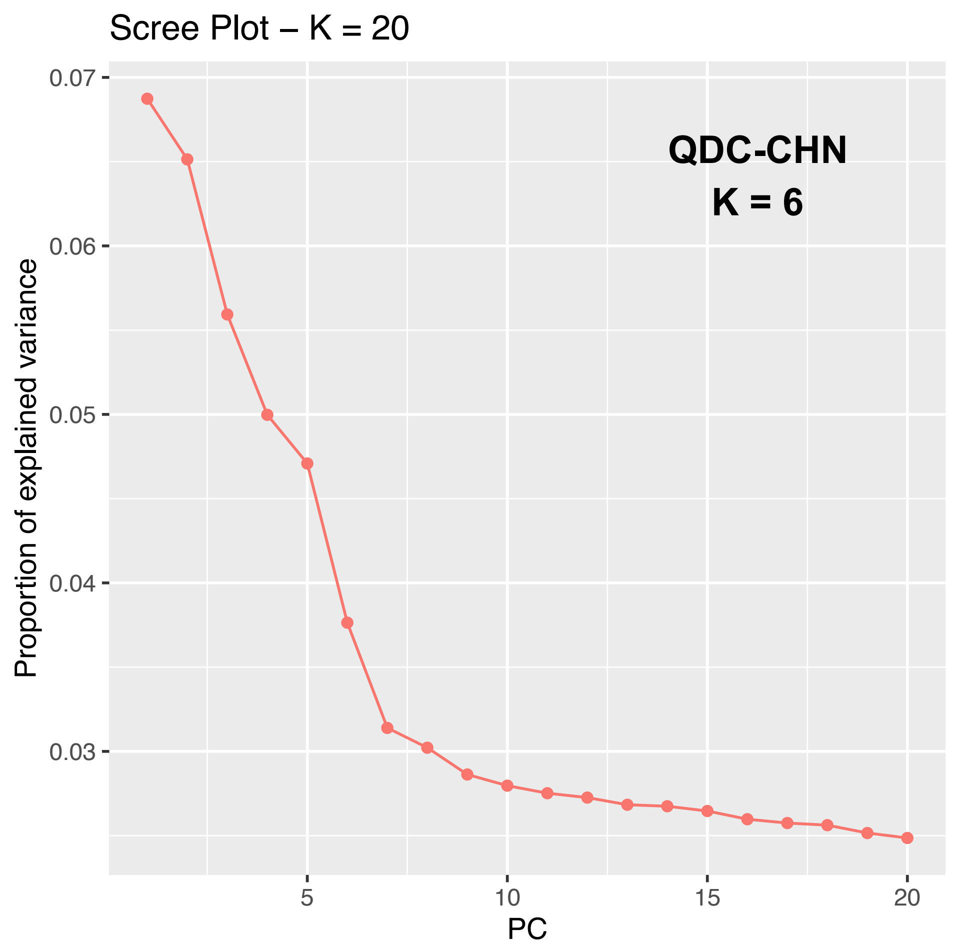

Scree Plot – K = 20
QDC-CHN
K = 6
Proportion of explained variance
0.07
0.06
0.05
0.04
0.03
PC
5
10
15
20

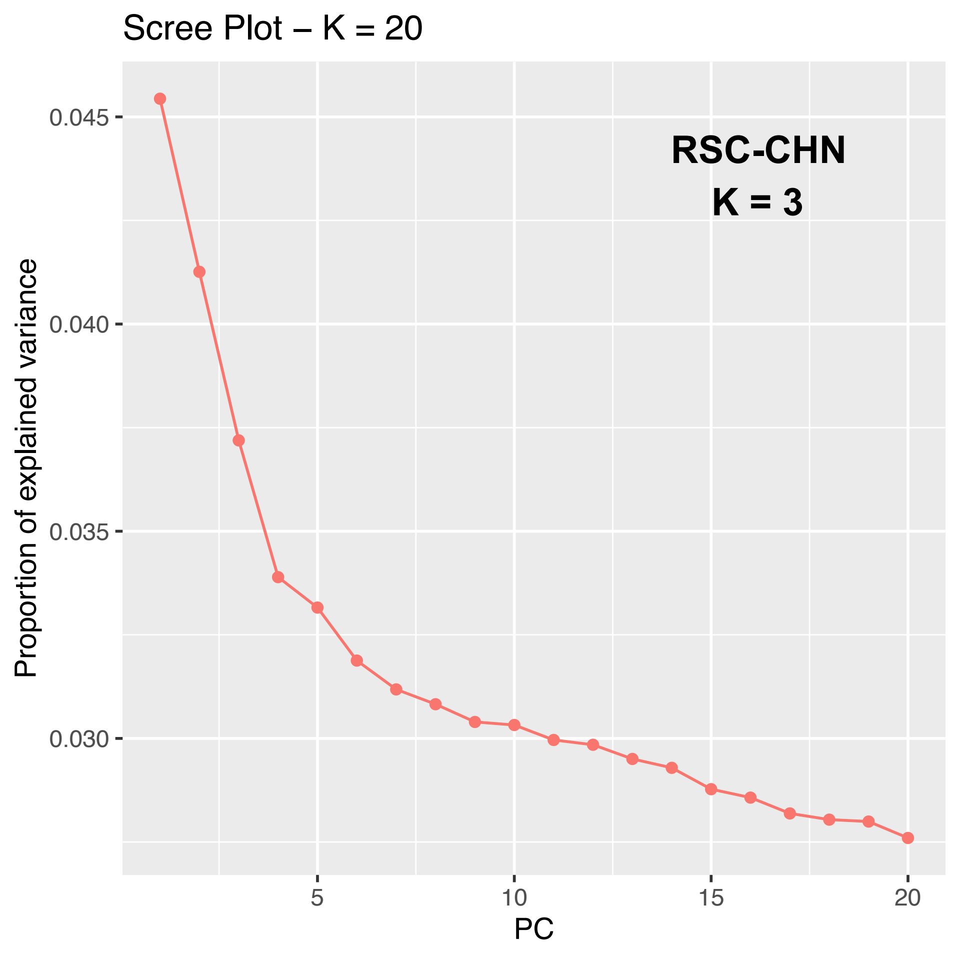
Scree Plot – K = 20
RSC-CHN
K = 3
Proportion of explained variance
0.045
0.040
0.035
0.030
PC
5
10
15
20

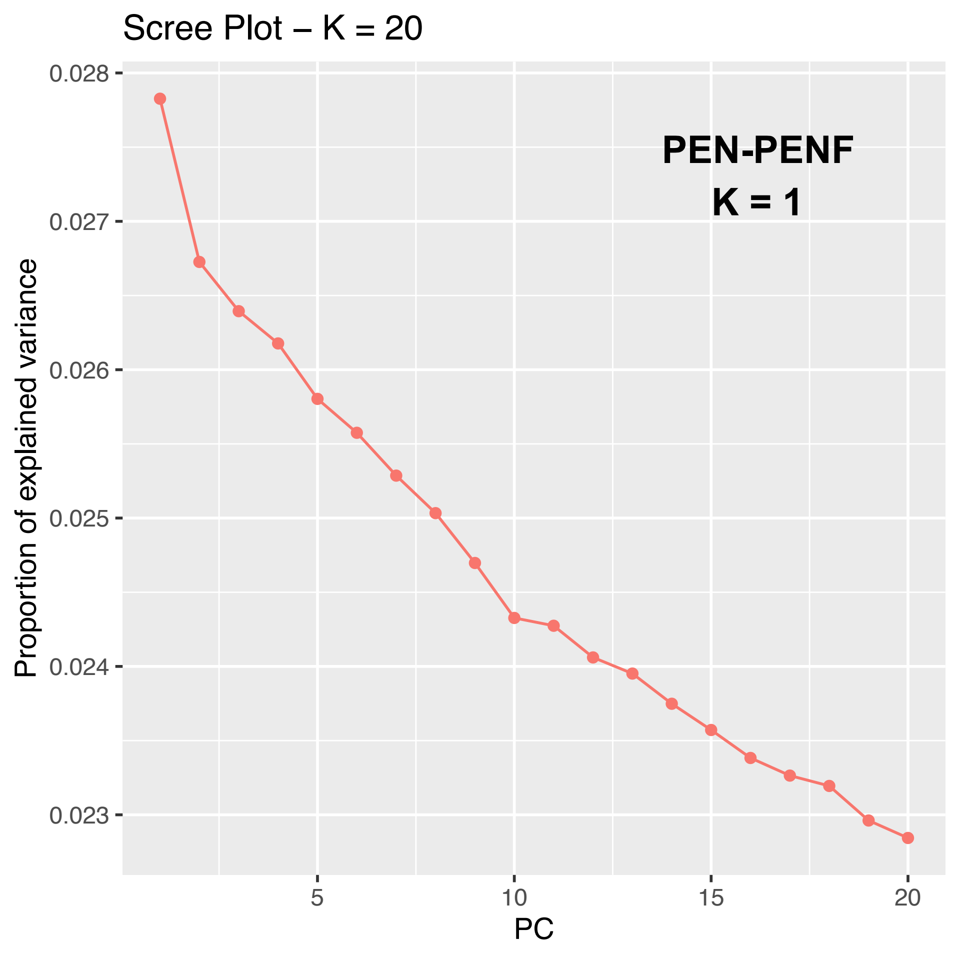
Scree Plot – K = 20
PEN-PENF
K = 1
Proportion of explained variance
0.028
0.027
0.026
0.025
0.024
0.023
PC
5
10
15
20

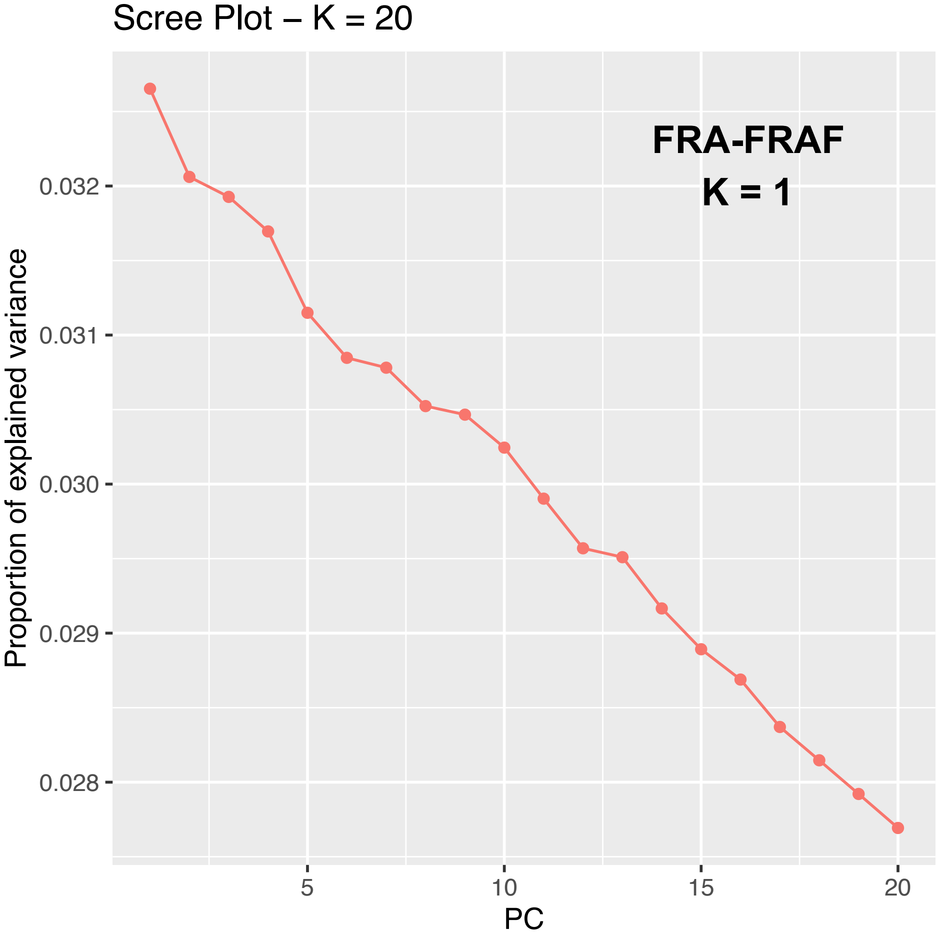
Scree Plot – K = 20
FRA-FRAF
K = 1
Proportion of explained variance
0.032
0.031
0.030
0.029
0.028
PC
5
10
15
20

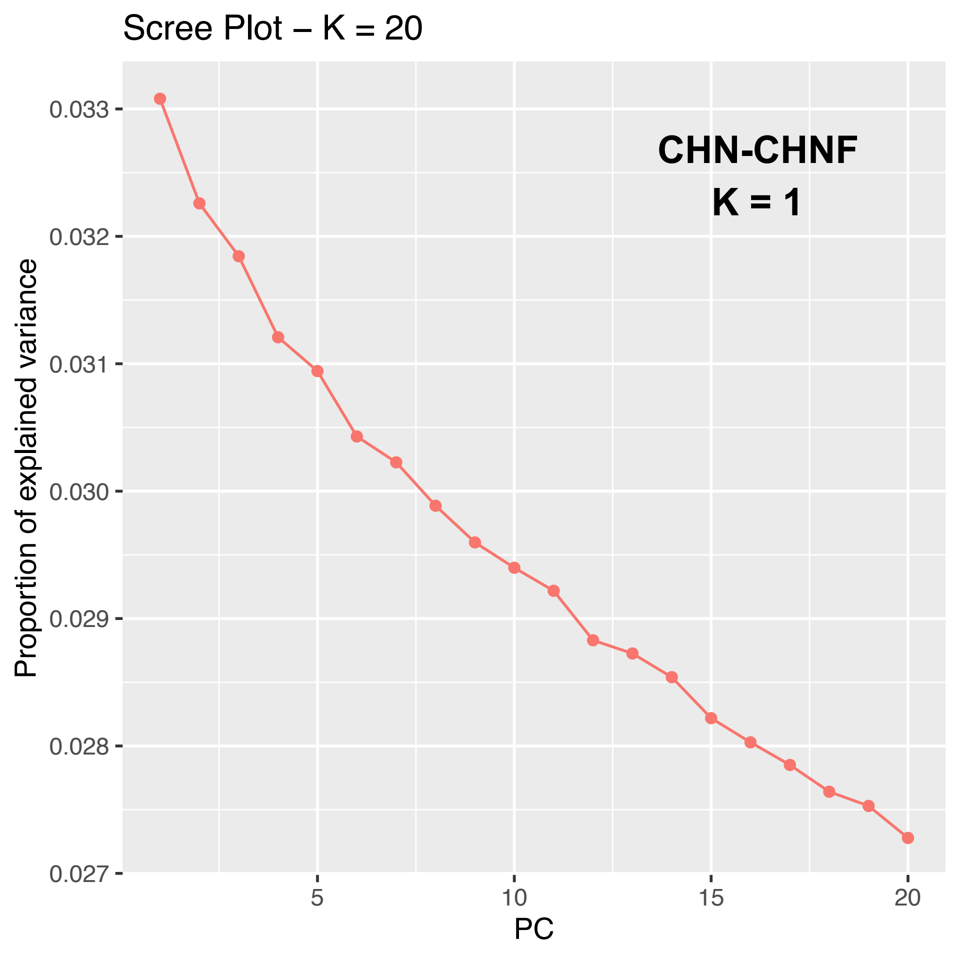
Scree Plot – K = 20
CHN-CHNF
K = 1
Proportion of explained variance
0.033
0.032
0.031
0.030
0.029
0.028
0.027
5
10
15
20
PC
