# Supplementary material for: Relative genomic impacts of translocation history, hatchery practices, and farm selection in Pacific oyster Crassostrea gigas throughout the Northern Hemisphere
Source: Evol Appl. 2020 Apr 17;13(6):1380–99. doi: 10.1111/eva.12965 (PMC7359842; doi:10.1111/eva.12965)
Supplement: Supplementary file 3 — Appendix S2 [file EVA-13-1380-s003.pdf]

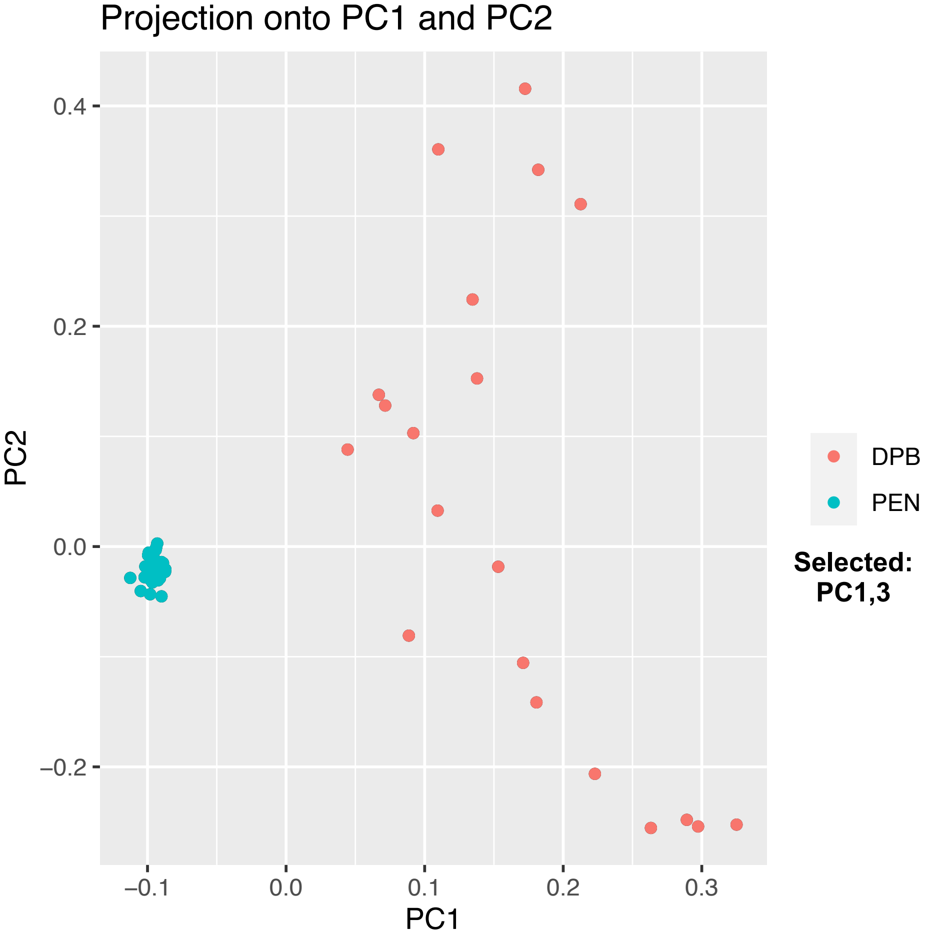

Projection onto PC1 and PC2
PC2
PC1
0.4
0.2
0.0
−0.2
−0.1
0.0
0.1
0.2
0.3
DPB
PEN
Selected:
PC1,3

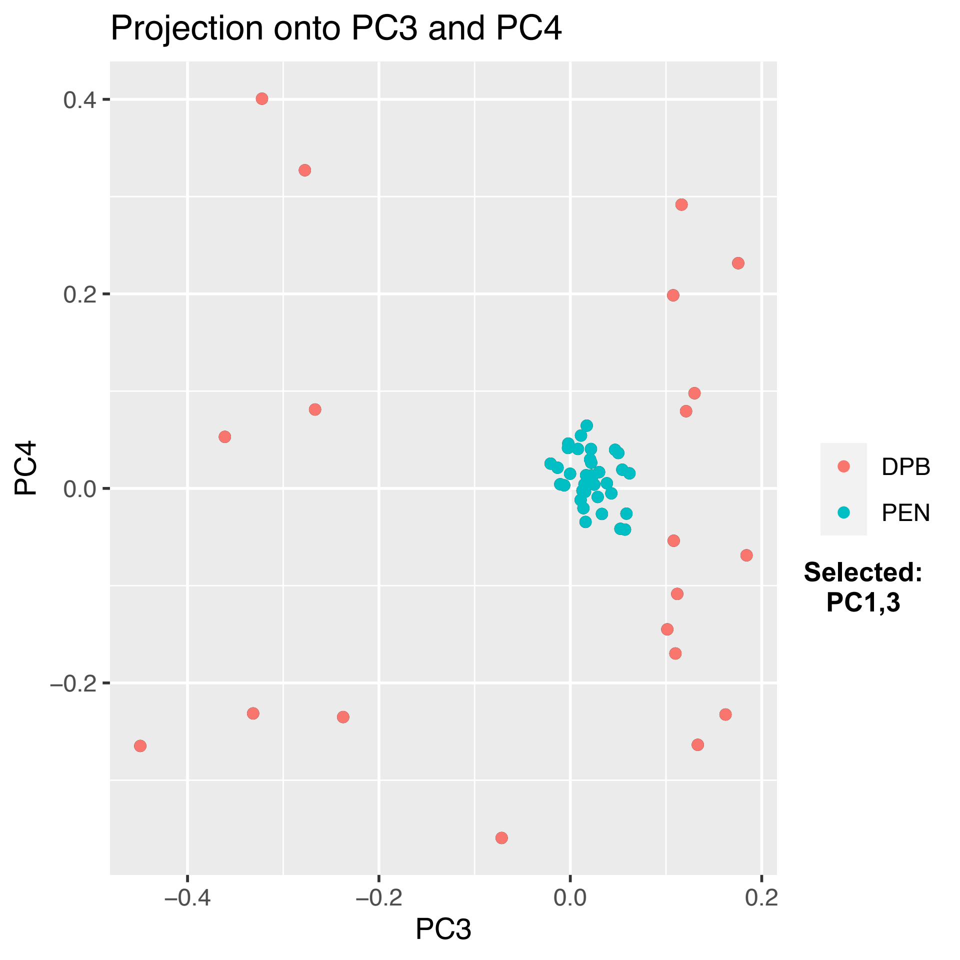
Projection onto PC3 and PC4
PC4
0.4
0.2
0.0
−0.2
PC3
−0.4
−0.2
0.0
0.2
DPB
PEN
Selected:
PC1,3

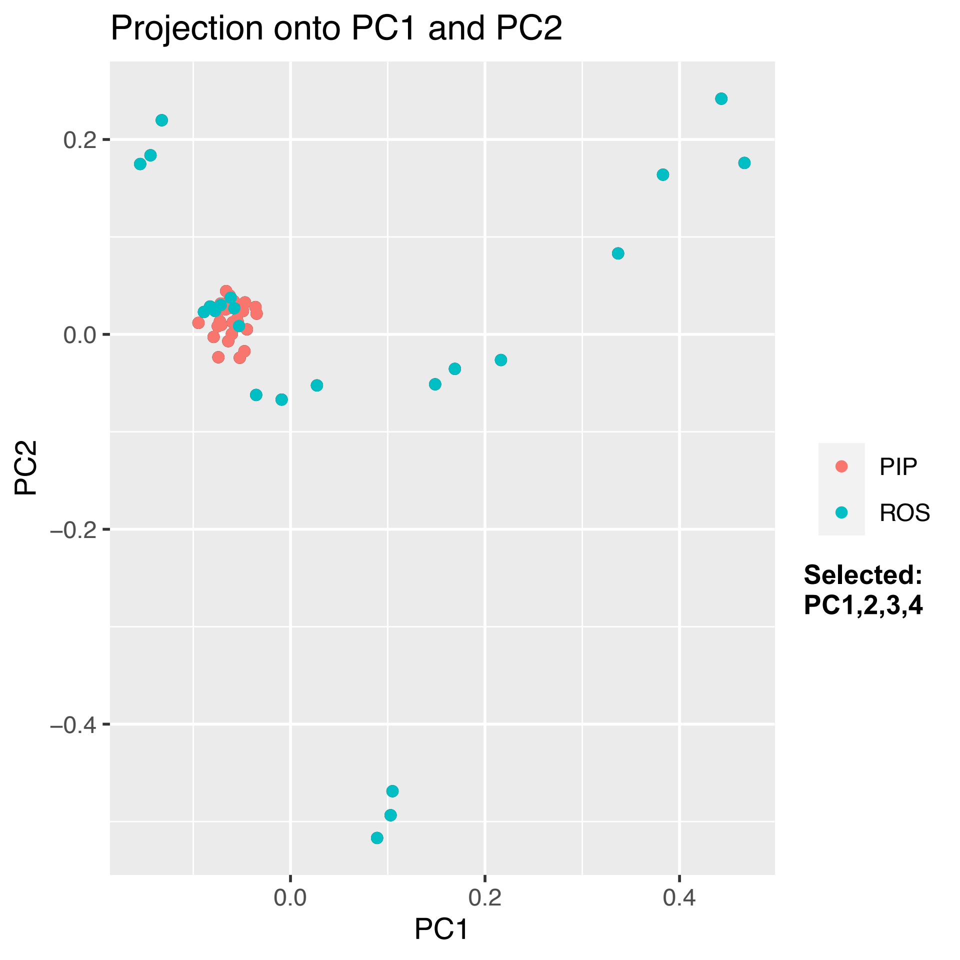
Projection onto PC1 and PC2
0.2
0.0
−0.2
−0.4
PC2
0.0
0.2
0.4
PC1
PIP
ROS
Selected:
PC1,2,3,4

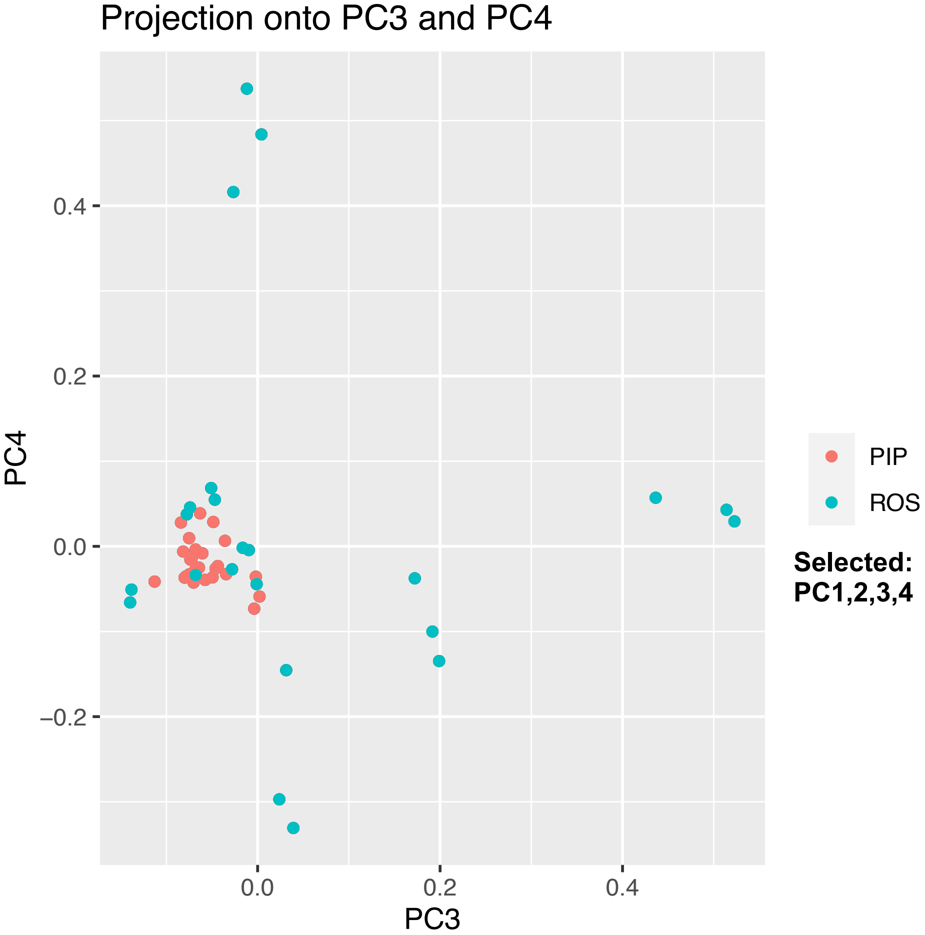
Projection onto PC3 and PC4
PC4
PC3
0.4
0.2
0.0
−0.2
0.0
0.2
0.4
PIP
ROS
Selected:
PC1,2,3,4

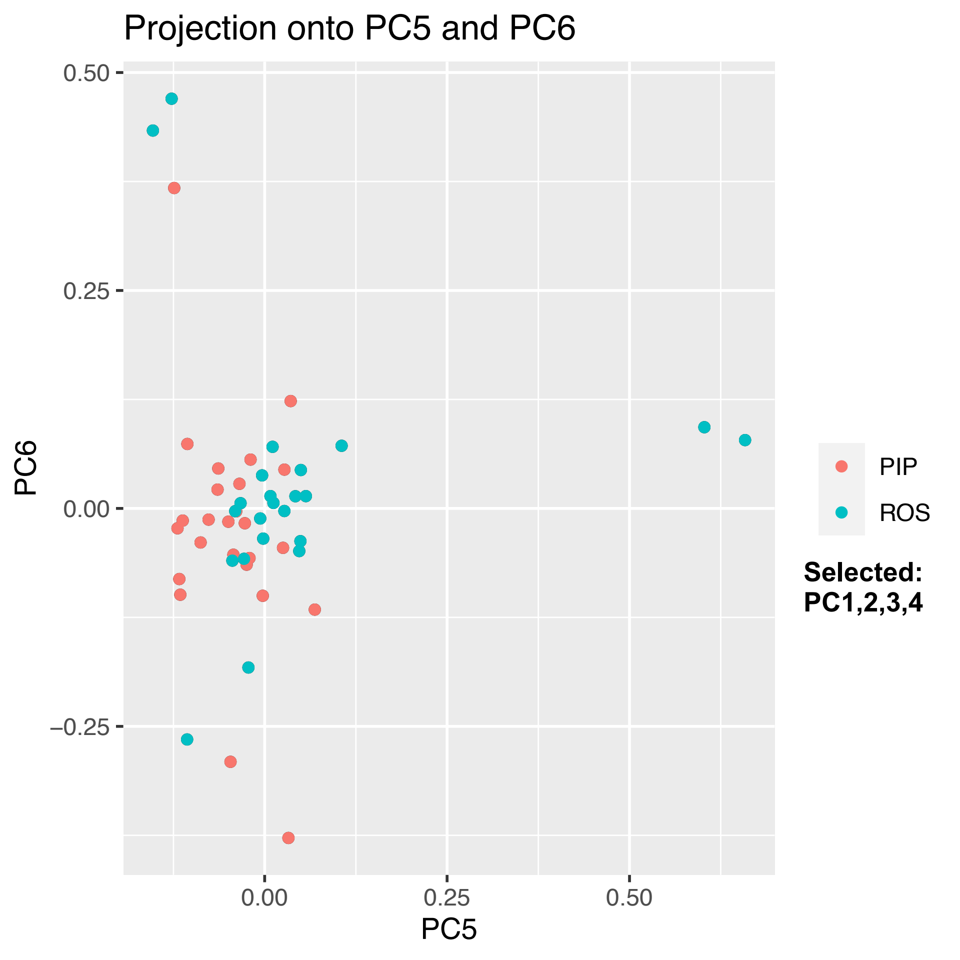
Projection onto PC5 and PC6
PC6
0.50
0.25
0.00
−0.25
0.00
0.25
0.50
PC5
PIP
ROS
Selected:
PC1,2,3,4

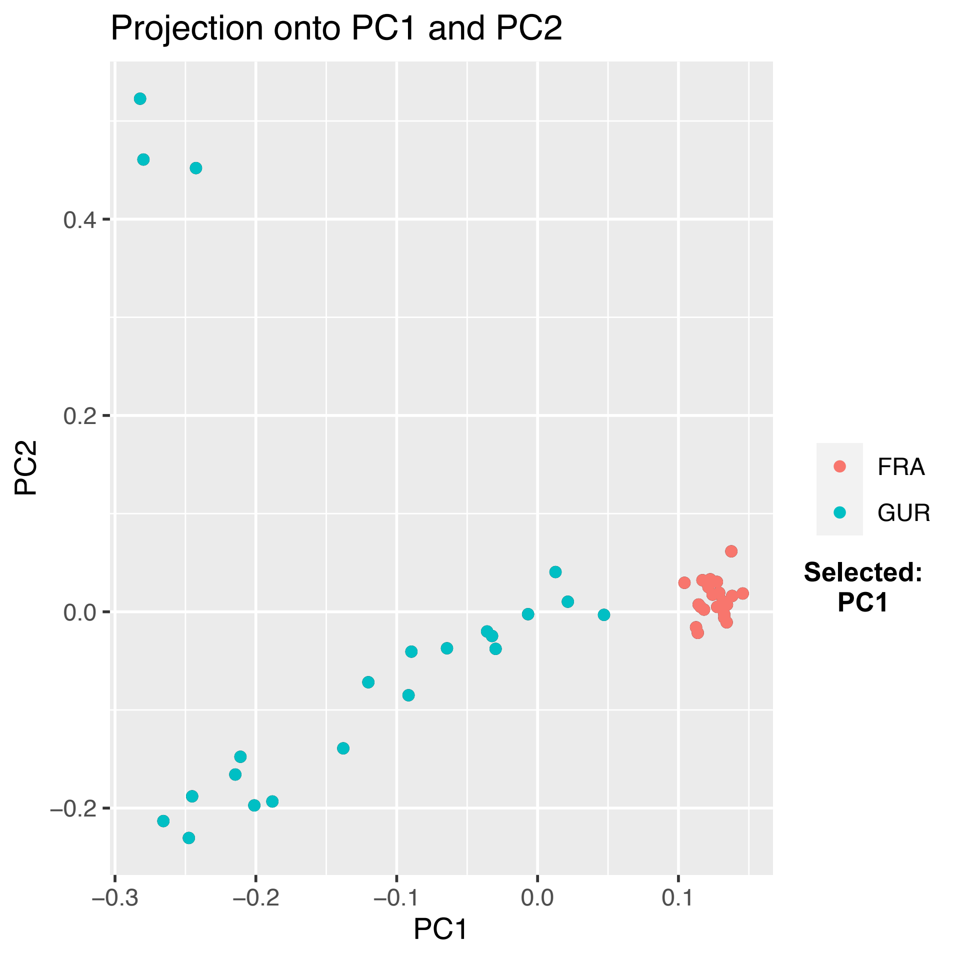
Projection onto PC1 and PC2
PC2
0.4
0.2
0.0
−0.2
PC1
−0.3
−0.2
−0.1
0.0
0.1
FRA
GUR
Selected:
PC1

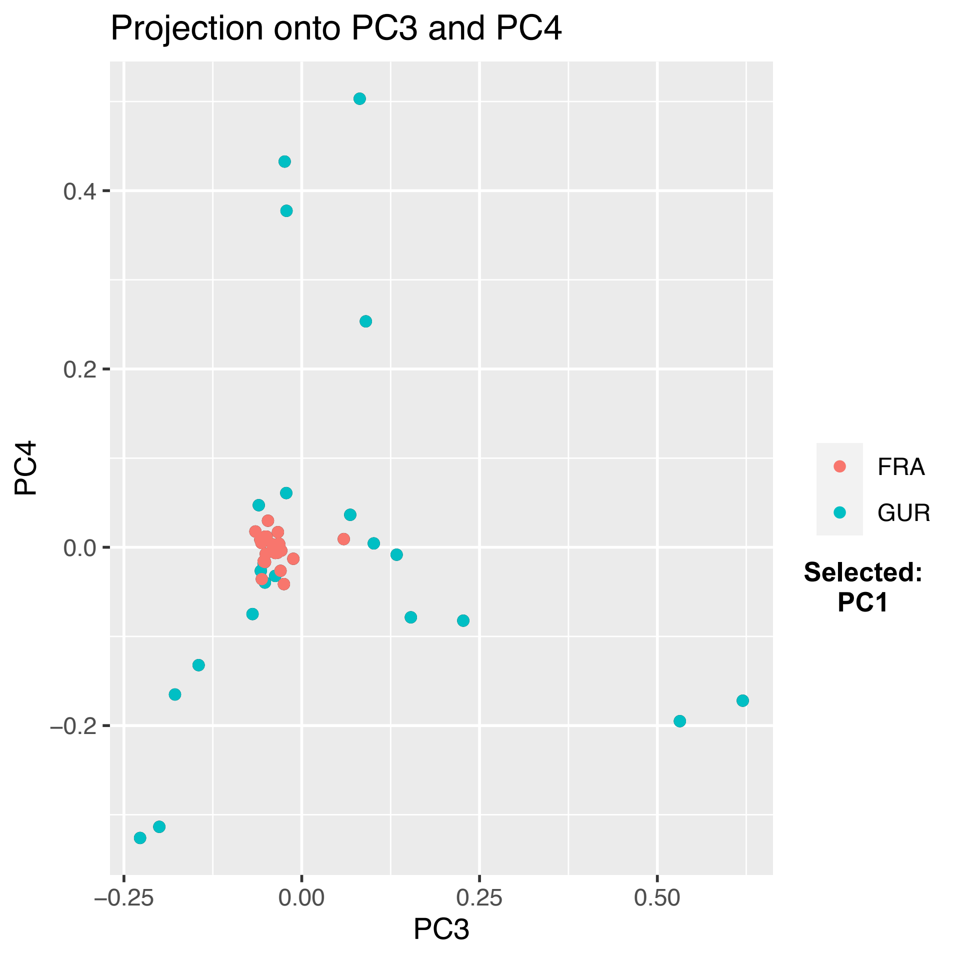

Projection onto PC3 and PC4
PC4
0.4
0.2
0.0
−0.2
PC3
−0.25
0.00
0.25
0.50
FRA
GUR
Selected:
PC1

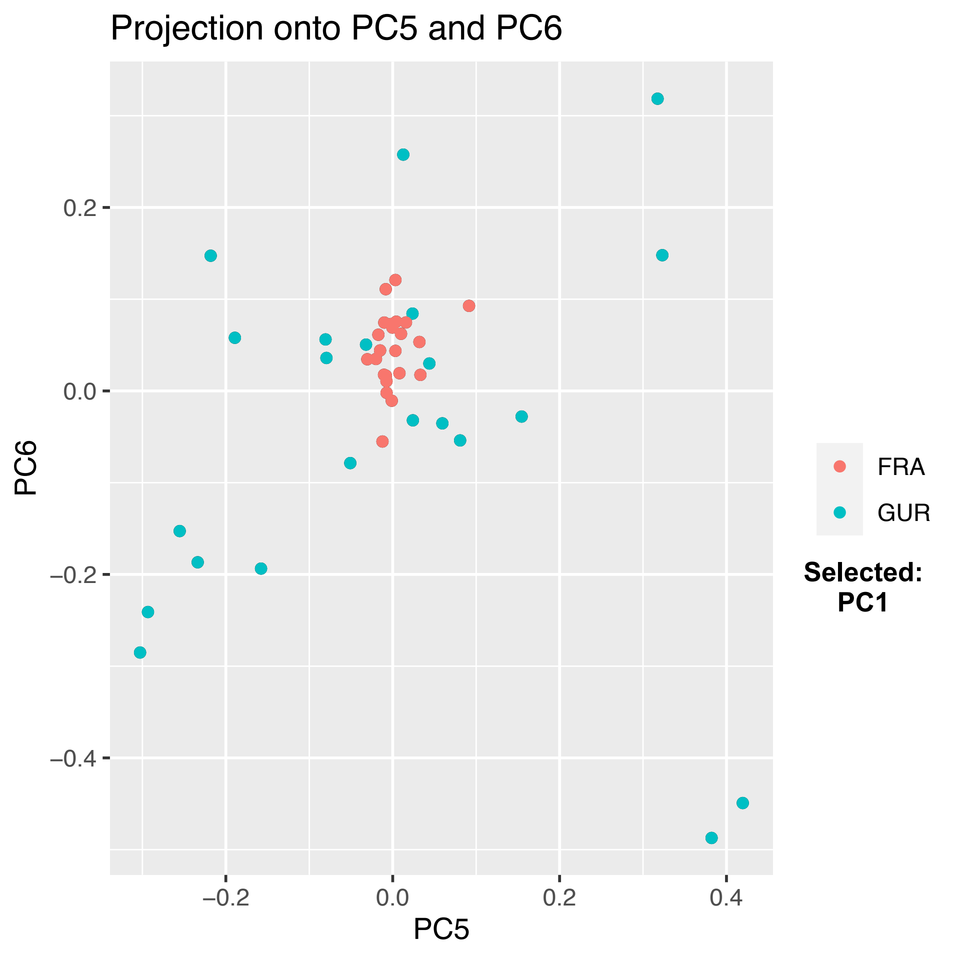
Projection onto PC5 and PC6
PC6
PC5
0.2
0.0
−0.2
−0.4
−0.2
0.0
0.2
0.4
FRA
GUR
Selected:
PC1

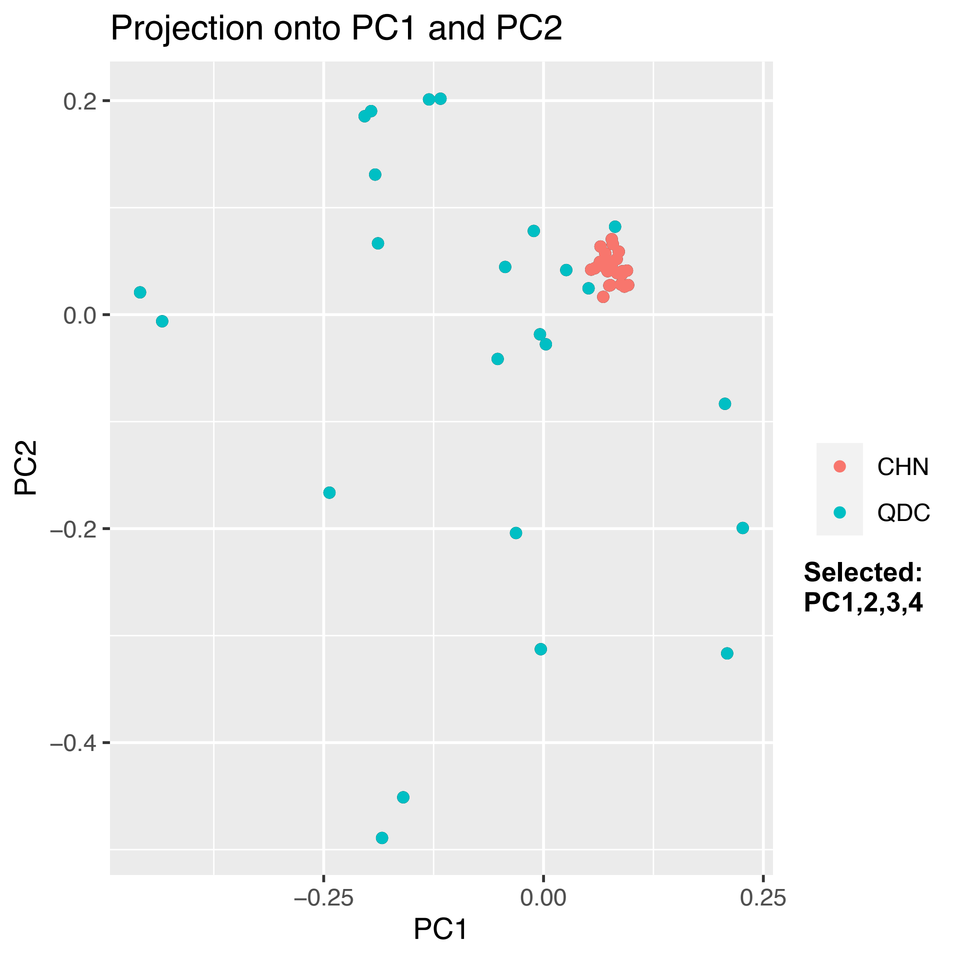

Projection onto PC1 and PC2
0.2
0.0
−0.2
−0.4
PC2
−0.25
0.00
0.25
PC1
CHN
QDC
Selected:
PC1,2,3,4

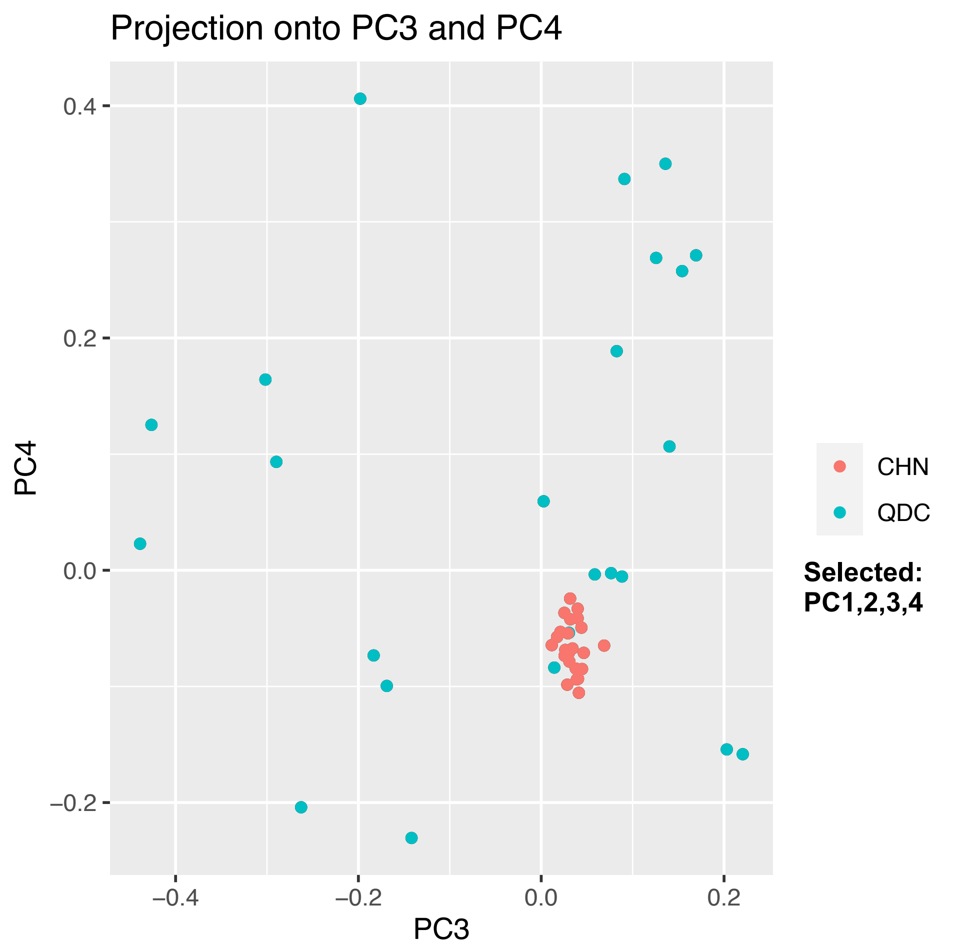

Projection onto PC3 and PC4
PC4
PC3
0.4
0.2
0.0
−0.2
−0.4
−0.2
0.0
0.2
CHN
QDC
Selected:
PC1,2,3,4

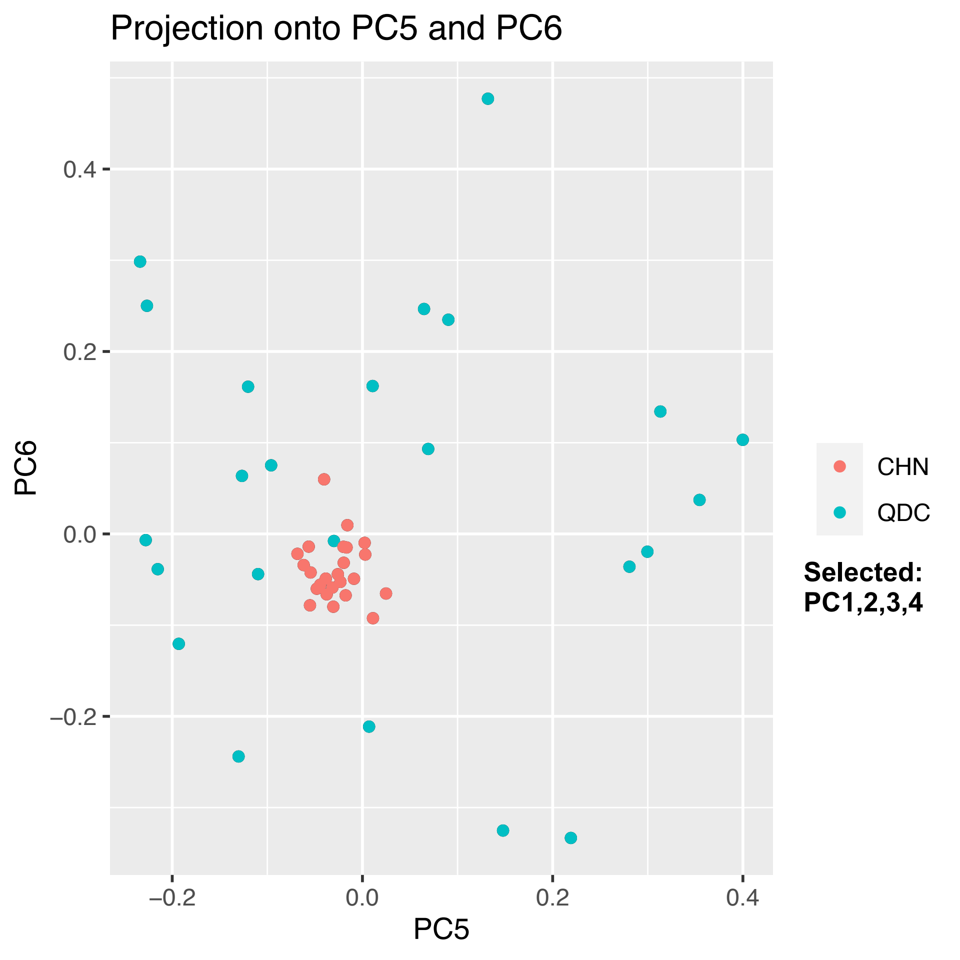
Projection onto PC5 and PC6
PC6
0.4
0.2
0.0
−0.2
PC5
−0.2
0.0
0.2
0.4
CHN
QDC
Selected:
PC1,2,3,4

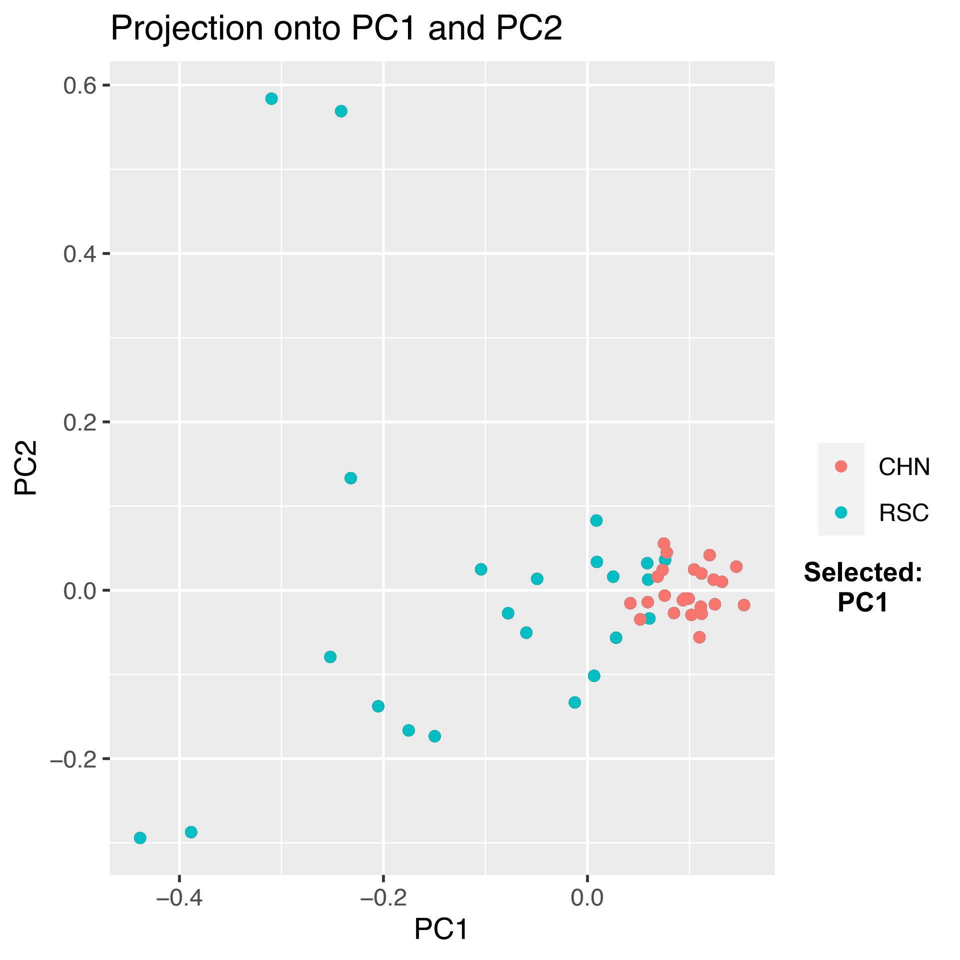
Projection onto PC1 and PC2
0.6
0.4
0.2
0.0
−0.2
PC2
−0.4
−0.2
0.0
PC1
CHN
RSC
Selected:
PC1

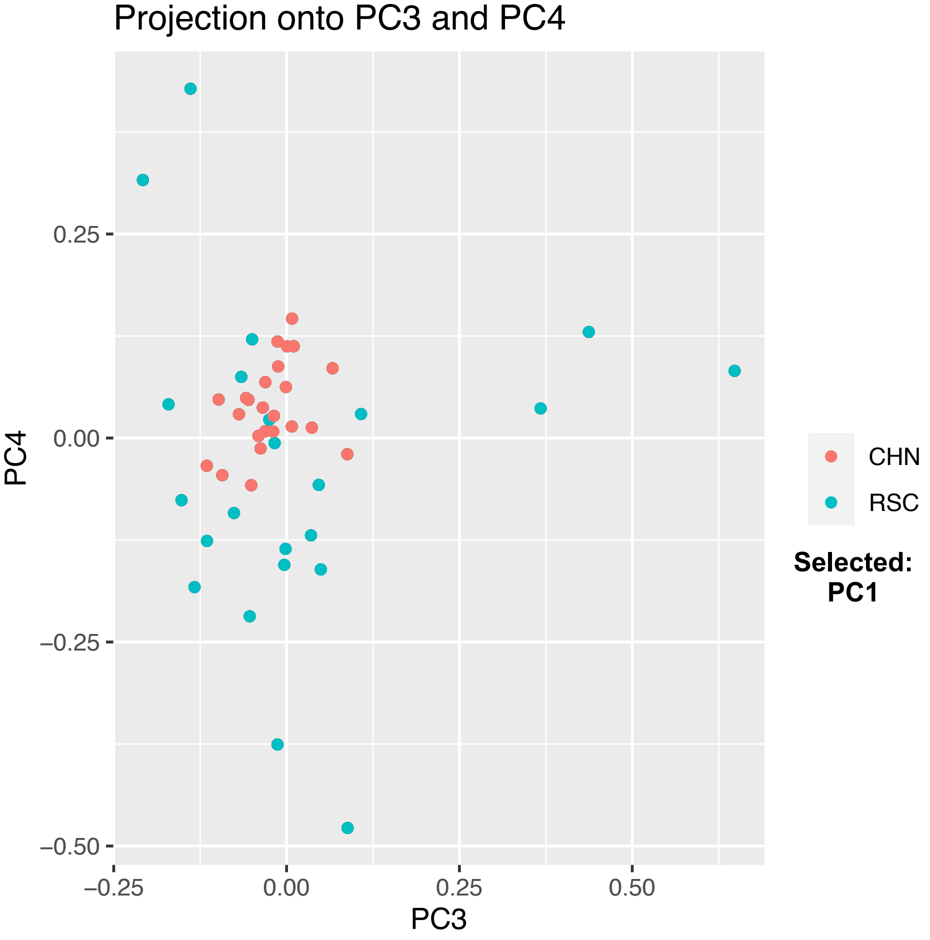

Projection onto PC3 and PC4
PC4
0.25
0.00
−0.25
−0.50
−0.25
0.00
0.25
0.50
PC3
CHN
RSC
Selected:
PC1

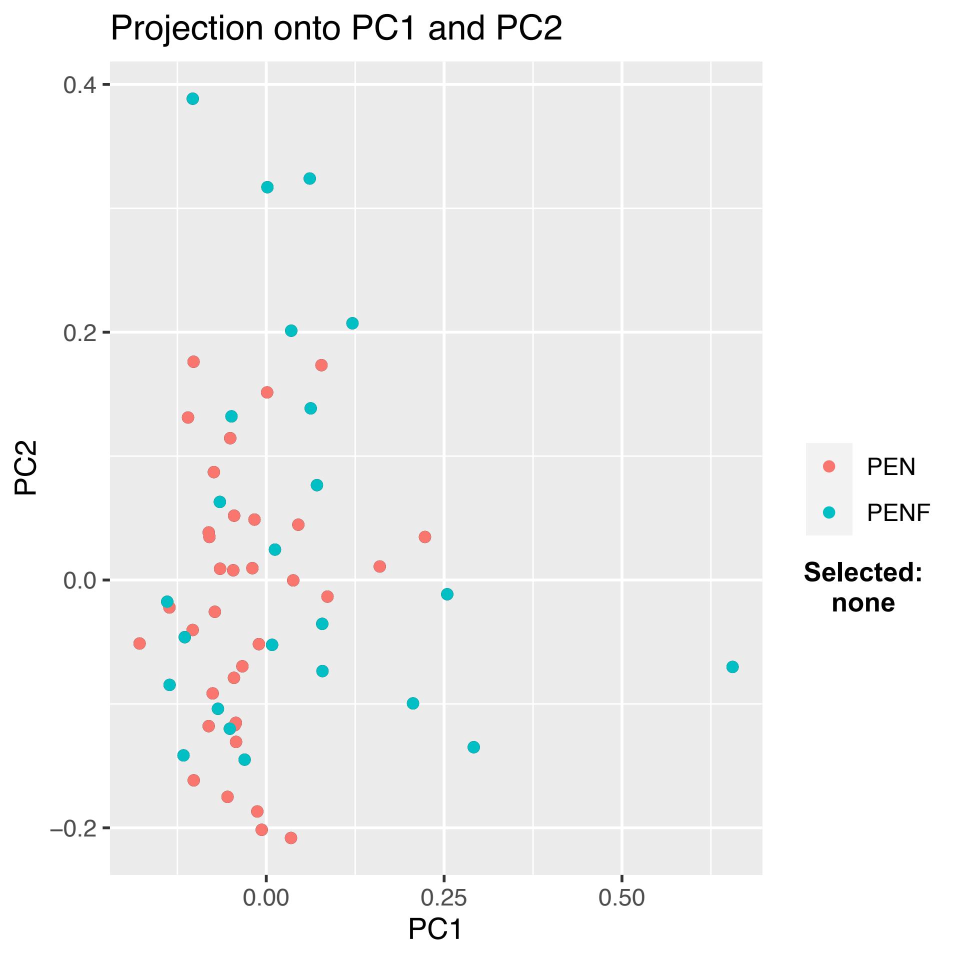
Projection onto PC1 and PC2
0.4
0.2
0.0
−0.2
PC2
0.00
0.25
0.50
PC1
PEN
PENF
Selected:
none

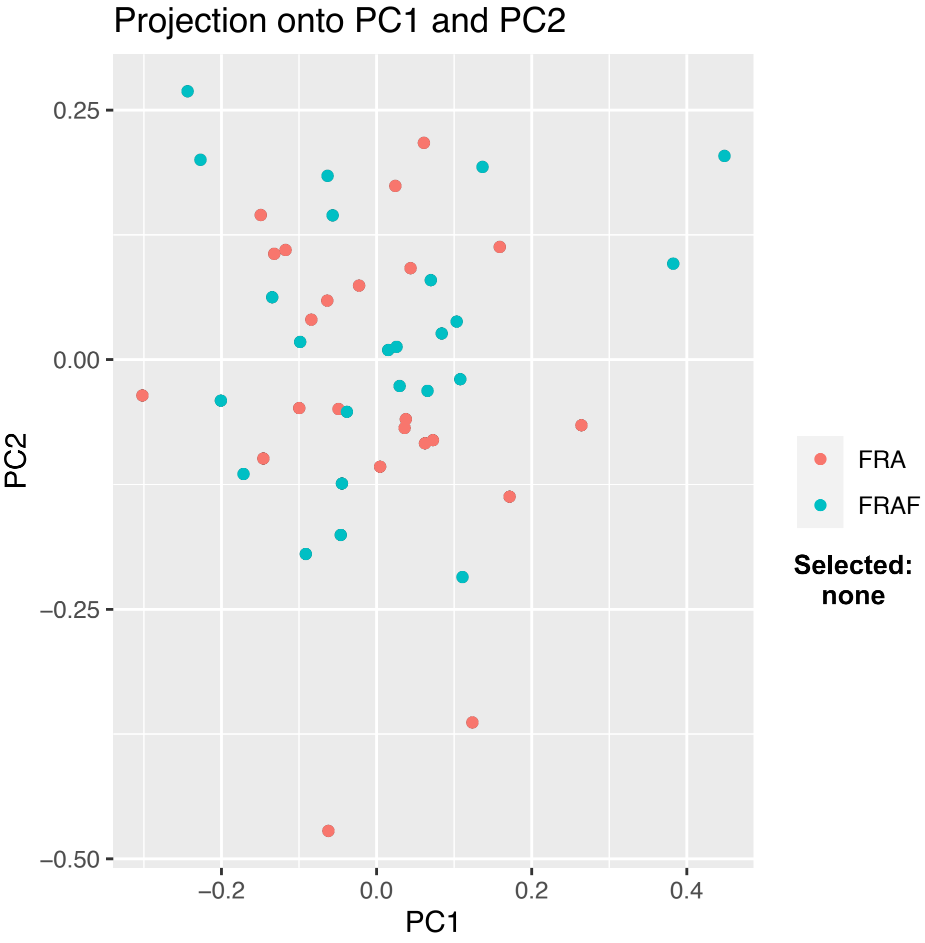
Projection onto PC1 and PC2
0.25
0.00
−0.25
−0.50
−0.2
0.0
0.2
0.4
PC1
PC2
FRA
FRAF
Selected:
none

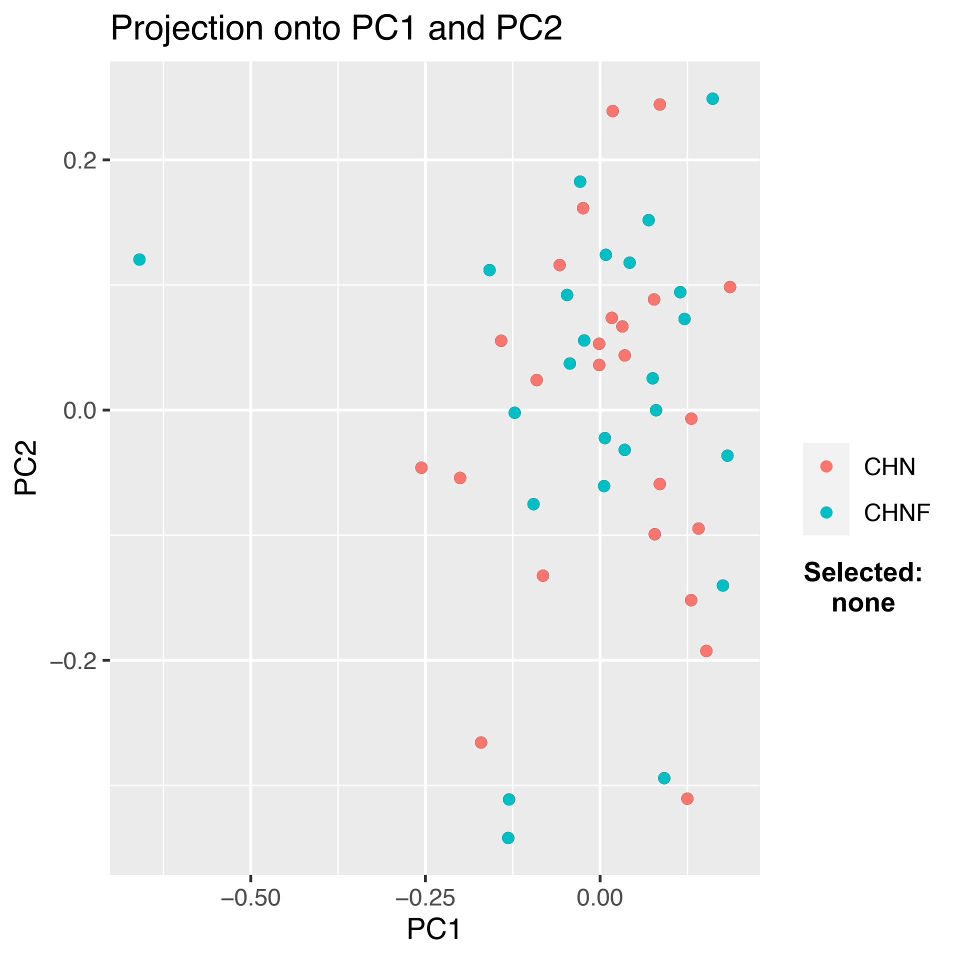
Projection onto PC1 and PC2
0.2
0.0
−0.2
−0.50
−0.25
0.00
PC1
PC2
CHN
CHNF
Selected:
none
